# Chemical Characterization of Seasonal PM_2.5_ Samples and Their Cytotoxicity in Human Lung Epithelial Cells (A549)

**DOI:** 10.3390/ijerph17124599

**Published:** 2020-06-26

**Authors:** Ao Di, Yun Wu, Mindong Chen, Dongyang Nie, Xinlei Ge

**Affiliations:** 1 Collaborative Innovation Center of Atmospheric Environment and Equipment Technology, Jiangsu Key Laboratory of Atmospheric Environment Monitoring and Pollution Control, School of Environmental Science and Engineering, Nanjing University of Information Science & Technology, Nanjing 210044, China; dioinuist@163.com (A.D.); caxinra@163.com (X.G.); 2 School of Atmospheric Sciences, Nanjing University, Nanjing 210023, China; dynie@nju.edu.cn

**Keywords:** fine particulate matter, chemical components, reactive oxygen species, apoptosis, Nanjing

## Abstract

In order to study the toxicity of fine particulate matter (PM_2.5_) sourced from different seasons on human health, we collected PM_2.5_ samples quarterly from March 2016 to February 2017 in Nanjing, China. The component analysis results showed that high proportions of water-soluble organic carbon (WSOC), SO_4_^2−^, Ca^2+^ and Mg^2+^ were found in the summer samples, while high proportions of NO_3_^−^, NH_4_^+^ and heavy metals were observed in the spring and winter samples. Then human lung epithelial cells (A549) were exposed to the PM_2.5_ samples. The toxicological results indicated that reactive oxygen species (ROS) production in the spring and winter samples was higher than that in the summer and fall samples, which was related to the contribution of some heavy metals and inorganic ions (e.g., Pb and NO_3_^−^). However, the apoptosis rates of the cells showed the opposite seasonal changes as what the ROS did, which might be caused by the higher WSOC content in the summer. In addition, regression analysis also showed the importance of the PM_2.5_ components in ROS production and apoptosis. Particularly, Zn had the strongest correlation with ROS production (R = 0.863) and cell apoptosis (R = 0.675); thus, the specific toxicity of Zn in PM_2.5_ deserves further investigation. Our results could be beneficial for assessing the health risks and controlling the toxic components of PM_2.5_ in Nanjing.

## 1. Introduction

With the rapid development of the economy and society in China, a large number of particles produced by industrial activity, transportation and coal burning has caused serious air pollution problems, and poses great risks to human health [1]. The impact of fine particulate matter (PM_2.5_) on human health has received widespread attention in recent years. Epidemiological studies show that PM_2.5_ can not only reach the deepest part of the lungs through the respiratory tract, enter alveoli and form deposits, but also penetrate the blood–gas barrier and enter into the blood circulation [2]. After that, PM_2.5_ can cause damage or dysfunction in vascular endothelial cells, and induce cardiovascular diseases such as atherosclerosis [3]. Animal models and cell studies prove that PM_2.5_ can induce oxidative damage and inflammatory reactions in the lungs [4,5,6]. In addition, PM_2.5_ can increase membrane permeability, facilitate the release of Cytochrome-C and the formation of apoptosis bodies, lyse the downstream apoptosis execution protein, destroy cells and eventually cause apoptosis [7]. 

PM_2.5_ consists of a carbonaceous core and various absorbed chemical components, such as metals, organic compounds, salts and biological components [8]. Carbonaceous compositions account for a large proportion of PM_2.5_, and they can penetrate into the lungs and cause respiratory tract inflammation [9]. Although metal elements are present in relatively small amounts, they are also non-negligible toxic components in PM_2.5_ due to their non-degradability, bioaccumulation and high toxicity [10]. Water-soluble ions (e.g., SO_4_^2−^, Cl^−^ and NH_4_^+^) are the main chemical components in PM_2.5_. In addition, they can change the pH of the exposed solution and affect cell viability [11,12]. It has been widely demonstrated that the composition of PM_2.5_ is closely related to its toxicity [13]. Epidemiological studies have found that carbonaceous composition exposure could affect the human cardiovascular system, respiratory system, nervous system and other aspects [14]. It also has been showed that an increase in the proportion of sulfate and nitrate in PM_2.5_ would lead to a relative increase in mortality [15]. As for the metal components, Cd and As adsorbed by PM_2.5_ from traffic sources could damage the human kidney [16]. Zn, Ni, Si, Ti and elemental carbon in PM_2.5_ were the primary factors that induced adverse pregnancy outcomes (e.g., a low birth weight) [17,18].

In recent years, many studies have shown that the main cause of PM_2.5_-induced lesions involved reactive oxygen species (ROS) [19]. ROS is chemically active and has a wide range of effects. Not only can it directly cause oxidative damage to biochemical macromolecules (e.g., nucleic acids and proteins), lead to DNA cross-linking, fragmentation and DNA–Protein crosslink formation, but it can also cause lipid membrane peroxidation, lead to cell dysfunction and then apoptosis [20,21]. Apoptosis is an orderly death of cells controlled by genes in order to maintain the stability of the internal environment [22]. It involves a series of gene activation, expression and regulation. Therefore, both ROS and apoptosis are considered as important endpoint indicators of toxicity. Many studies have showed the relationship between oxidative damage or cell death with different chemical components of PM_2.5_ [13]. Metal elements, such as the Fe, V, Cu and Mn contained in PM_2.5_, can promote the production of intracellular ROS through the Fenton reaction, and then impair antioxidant enzyme function [23,24]. Some water-soluble components (SO_4_^2−^ and NH_4_^+^) were found to be significantly correlated with ROS production (*p <* 0.05, R *>* 0.70) [25]. However, due to the diversity in PM_2.5_ composition, its toxicity varies greatly and no conclusion has been reached at present. Temporal–spatial-specific analyses are necessary to understand the PM composition and variability for regional air pollution control.

As the capital of Jiangsu Province, Nanjing has experienced rapid economic development in recent years. Industrial coal combustion and motor vehicle exhaust emissions have been intensified, which brought tremendous pressure on the atmospheric environment. Most studies regarding PM_2.5_ focused on the physical and chemical characteristics of PM_2.5_, but the toxic effects of PM_2.5_ in this area are still not clear. Generally, emission sources and atmospheric conditions dictate the chemical composition of PM_2.5_ in Nanjing, resulting in seasonal differences in PM_2.5_ composition, which will further affect its toxic properties [26,27]. For example, the inhibition of cell viability caused by PM_2.5_ was higher in summer samples [28]. In this study, PM_2.5_ samples were collected from the northern suburbs of Nanjing in four seasons of 2016 to determine the variations in its components, including water-soluble organic carbon (WSOC), water-soluble ions and heavy metal elements. Simultaneously, the degree of oxidative damage and apoptosis in human lung epithelial cells A549 were examined. Our results could provide a scientific basis for regional health risk assessment and health protection measure formulation.

## 2. Materials and Methods

### 2.1. Reagents

A human lung epithelial cell line (A549) was obtained from the school of Public Health, Nanjing Medical University (Nanjing, China). RPMI-1640 medium and phosphate buffered saline (PBS) were purchased from Bio-Channel (Nanjing, China). Fetal bovine serum (FBS) was from Gibco (New Castle, Australia). The 2’,7’-dichlorofluorescein diacetate (DCFH-DA) was obtained from Sigma-Aldrich (St Louis, MO, USA). Annexin V-FITC/PI Apoptosis Detection Kit was purchased from Beyotime Bio-technology (Shanghai, China).

### 2.2. PM_2.5_ Collection and Preparation

Urban atmospheric particulate matter PM_2.5_ was collected during the four seasons of 2016 (from March 2016 to February 2017). Twenty samples from the middle month (April, July, October and January) of each quarter were selected to represent the different seasons. The sampling site was located on the campus of Nanjing University of Information Science and Technology (NUIST), China. Particles were obtained on quartz filters (203 mm × 254 mm, Whatman, Maidstone, UK) using a high-volume air sampler (Tisch Environmental, Cleves, OH, USA) [29]. The collection flow rate was 1130 L/min (24 h).

**Figure 1:**
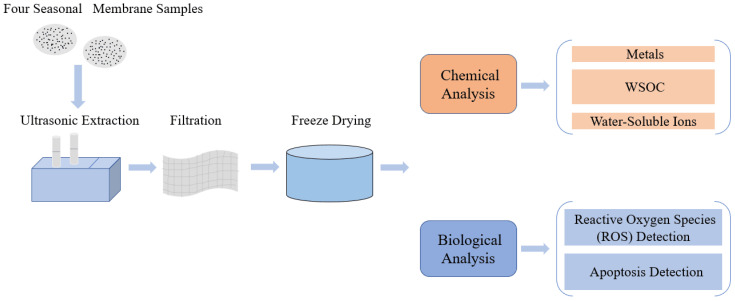
Schematic of the experimental design. WSOC: water-soluble organic carbon.

Quartz filters containing PM_2.5_ were cut into small strips and placed into ultrapure water. The samples were treated using ultrasonic extraction in an ice bath for 20 min and repeated 3 times to extract the PM_2.5_ [28,30]. Different filters of the same season were extracted, vacuum freeze-dried and pooled to have a homogeneous batch of particles, representative of the sampling season. Part of the dried samples were suspended in a phosphate-buffered saline (PBS) solution and stored at 4 °C for the cytotoxicity tests. Other dried samples were stored at −80 °C. Figure 1 shows the schematic of the experimental design.

### 2.3. PM_2.5_ Composition Analysis

After freeze-drying, particles were digested with 5 mL HNO_3_ in a microwave digestion instrument (GEM Corpooration, Charlotte, NC, USA). The digested solution was filtered, and diluted to 50 mL with ultrapure water. Heavy metal elements (As, Zn, Pb, Co, Cd, Ni, Mn, Cr, Cu and Al) were then detected by inductively coupled plasma mass spectrometry (ICP-MS, Thermo Fisher Scientific, Waltham, MA, USA). Two standard reference materials (GBW07403 and GBW08401) and a blank filter were analyzed in parallel with the samples. The recovery rates of all the elements were within 100 ± 15% [29].

Meanwhile, the other dry samples from each season were dissolved with 45 mL of ultrapure water and treated using ultrasonic extraction in an ice bath for 20 min. The solution was then filtered with a 0.22 µm filter to remove any particles. The cations in the solution were detected by ICS-3000 Multi Ion Chromatography (Dionex, Sunnyvale, CA, USA) equipped with the Ion Pac CS16-type separation column; the anions were detected by ICS-2000 Ion Chromatography (Dionex, Sunnyvale, CA, USA) equipped with the Ion Pac AS11-HC-type separation column. The methanesulfonate elution and KOH elution methods were used to analyze the cations and anions, respectively [31]. The total water-soluble organic carbon (WSOC) was detected using the LIGUE TOC-ANALYZER Organic Carbon Analyzer (Elementar, Hanau, Germany) [32].

### 2.4. Cell Culture and Exposure

A549 cells were cultured in RPMI-1640 medium supplemented with 10% FBS. They were maintained in a humidified incubator at 37 °C with 5% CO_2_. Logarithmic growth cells were used throughout this study. In each exposure experiment, A549 cells were seeded in six-well plates at a density of 5 × 10^5^ cells/well. Then cells were exposed to different concentrations of PM_2.5_ (0, 50, 100, 200 and 400 μg/mL) for 24 h. These concentrations were selected according to our preliminary experiments with the PM_2.5_ concentrations ranging from 0 to 1000 μg/mL. There were three parallels for each concentration.

### 2.5. ROS and Apoptosis Assay

Cellular ROS levels were assessed using the fluorescent probe DCFH-DA [33]. After 24 h of exposure, DCFH-DA was diluted at a ratio of 1:1000 with serum-free medium, and the diluent was added to label the exposed cells. The cells were maintained at 37 °C in the dark for 30 min. After washing with PBS for 2–3 times, cells were collected and detected by flow cytometry (Beckman, Miami, FL, USA). The wavelength of excitation and emission were 480 nm and 525 nm, respectively.

Cellular apoptosis was measured using the Annexin V-FITC/PI kit according to the manufacturer’s protocol. Briefly, the exposed cells were harvested, washed and resuspended in a buffer solution at a density of 1 × 10^5^ cells/well. A total of 5 µL of Annexin V-FITC and 10 µL of PI were added into each well. The treated cells were incubated in the dark at room temperature for 20 min. Afterwards, they were collected and detected by flow cytometry. FITC and PI fluorescence were both excited at 488 nm, while they were collected at 525 nm and 630 nm, respectively.

### 2.6. Statistical Analysis

To quantify the relationships between the PM_2.5_ compositions and toxic endpoints, regression analysis was performed between the heavy metals, water-soluble ions, WSOC, intracellular ROS and apoptosis rates. A t-test and one-way ANOVA were used to evaluate the differences among the treatments (SPSS 12.0) (SPSS Inc., Chicago, IL, USA). Statistical significance was accepted when *p* < 0.05. 

## 3. Results and Discussion

### 3.1. PM_2.5_ Chemical Characteristics

**Figure 2:**
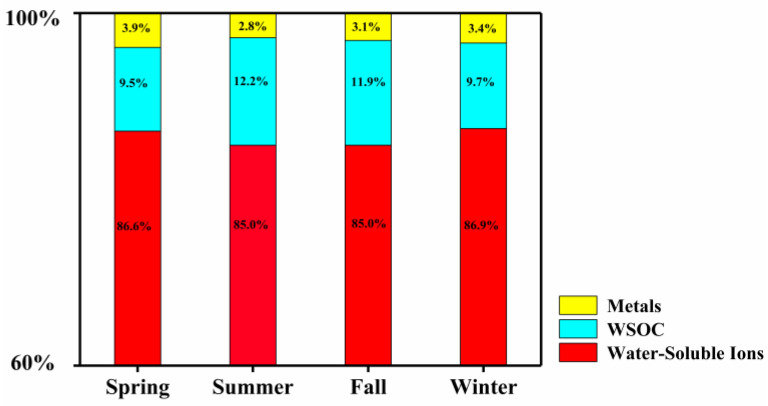
Relative percentages of different chemical components in PM_2.5_. WSOC: water-soluble organic carbon.

**Figure 3:**
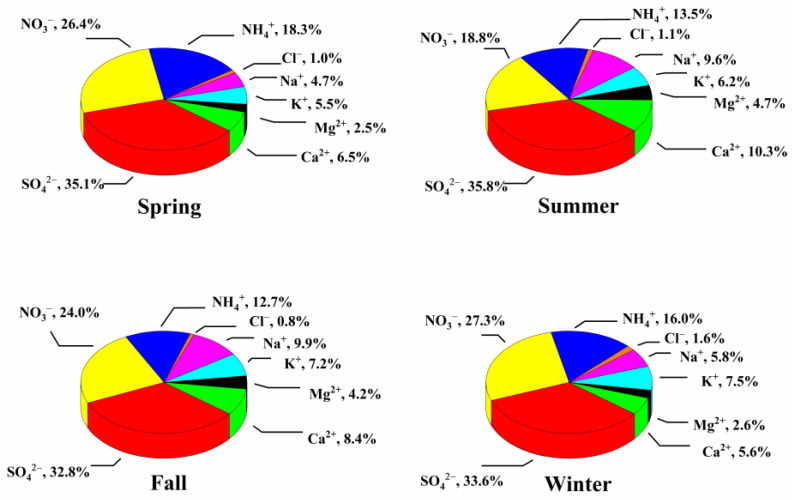
Relative percentages of each water-soluble ion to all water-soluble ions tested.

**Figure 4:**
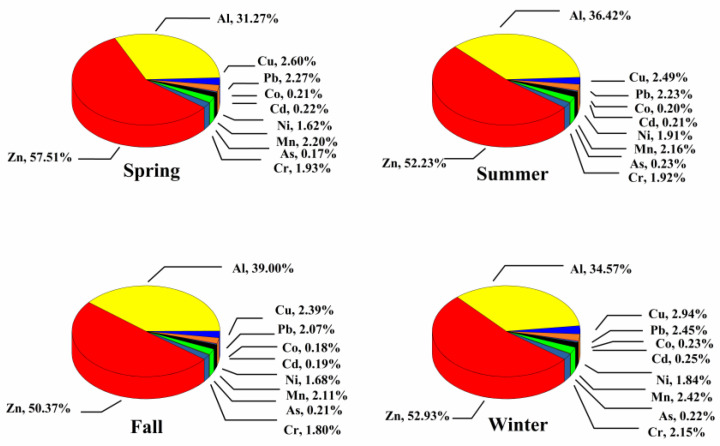
Relative percentages of each heavy metal to all heavy metals tested.

The major toxic components of PM_2.5_, including water-soluble ions (SO_4_^2−^, NO_3_^−^, NH_4_^+^, Na^+^, K^+^, Ca^2^^+^, Mg^2^^+^, Cl^−^), WSOC and heavy metal ions (As, Zn, Pb, Co, Cd, Ni, Mn, Cr, Cu, Al), were all detected in this study. Their relative proportions are shown in Figure 2, Figure 3 and Figure 4.

#### 3.1.1. WSOC

Organic carbon is an important part of organic aerosol, which accounts for 10–70% of the total PM_2.5_ mass [34]. A large proportion of organic carbon (20–60%) is water-soluble [35]. In this study, the WSOC sourced from the four seasons accounted for 9.5–12.2% of all species tested. Our previous measurement in Nanjing (May 2015 to May 2016) also showed a relatively low level of WSOC, which may be related to the emission sources [36]. Our results are comparable to those found in other cities, in that organic carbon made up 5.7–8.4% and 10.0% of the PM_2.5_ in Thessaloniki (Greece) and São Paulo (Brazil) [37,38], respectively. In addition, the WSOC had the highest proportion in the summer and fall, followed by winter and spring. Similar measurements were also reported in our previous study [28]. High temperatures and strong sunlight facilitate the production of secondary organic aerosols, which may explain the increased organic carbon proportions in the summer [39]. It should be noted that only the WSOC components were detected in this study; the specific species of carbon-containing organic matter in the PM_2.5_ have not been distinguished.

#### 3.1.2. Water-Soluble Ions

Water-soluble inorganic ions are another important chemical compositions of PM_2.5_. In this study, the percentage of water-soluble ions to total PM_2.5_ mass was the highest in winter (67.4%), followed by spring and summer (59.7% and 37.2%, respectively), and the lowest in fall (33.9%). This may be related to the unfavorable meteorological diffusion conditions in winter [40]. Further, these values in Nanjing were higher than those in Xiamen (28.7%), but comparable to those in Shenyang (55.9%) or Shanghai (55.9%) [41,42,43]. Since they come from different sources, their contents can be used to reflect the origin of the particles [38]. Generally, SO_4_^2−^ is a secondary pollutant produced from sulfur dioxide. NO_3_^−^ mainly comes from the conversion of its gaseous precursor, NOx. NH_4_^+^ is also an indicator of secondary pollution, and is mainly formed by the emission of NH_3_. Cl¯, K^+^ and Mg^+^ are often derived from the combustion emission of power plants and incinerators. Besides, K^+^ can be used as a marker of biomass combustion. Ca^+^ usually comes from soil sources, and it is often used as a marker of construction dust. 

SO_4_^2^^−^, NO_3_^−^ and NH_4_^+^ (collectively called SNA) were the major pollutants in PM_2.5_. SNA accounted for 68.1–79.8% of the total water-soluble ions, indicating that secondary pollution in this area was serious. Specifically, the proportion of SO_4_^2^^−^ was higher in the warmer seasons (spring and summer), which may be due to the enhanced oxidation of SO_2_ during the warm period. In contrast, the concentration of NO_3_^−^ was high in spring and winter but low in summer and fall. Under high temperature conditions, NH_4_NO_3_ is easily volatilized and converted to its gaseous precursors (NH_3_ and HNO_3_), resulting in relatively low NO_3_^−^ concentration in the summer. As expected, NH_4_^+^ had similar seasonal differences as NO_3_^−^. The ratio of NO_3_^−^ to SO_4_^2^^−^ (NO_3_^−^/SO_4_^2^^−^) can be used to distinguish the fixed sources (e.g., coal burning) from the mobile sources (e.g., vehicle exhaust). If the ratio is greater than 1, a mobile source would be the main factor, and vice versa. This ratio was 0.48–0.81 in our study, similar to the results of Zhou et al. (0.76) [44], indicating that the fixed source was still the main source of pollution in Nanjing. As for the cations, the content of K^+^ was relatively high in the winter, which may be related with winter coal emission or biomass combustion. Differently, Ca^2^^+^ and Mg^2^^+^ had relatively high amounts in the summer, which might be caused by the frequent construction and increased dust in summer.

#### 3.1.3. Heavy Metals

Different contents and species of metals have been found in PM_2.5_, which may be caused by different PM_2.5_ sources and environmental conditions [45]. Among all the measured metals, Al and Zn were the main elements in the PM_2.5_ samples of all four seasons, and accounted for more than 80% of the total metals. Al is a representative element of the crust source, while Zn mainly comes from fuel combustion and diesel engine emission [46]. Other elements can also indicate different sources, such as Pb is from coal combustion and motor vehicle exhaust; Cu is from coal combustion, industrial emission, motor vehicle tires and mechanical wear; and As is from coal-fired emissions, as well as metallurgical and chemical industries [47,48]. In this study, high proportions of Zn, Pb, As and Cu were found in PM_2.5_, which was related to the fact that our sampling site is located in a traffic pollution area near chemical plants. Further, the percentage of heavy metals in the spring and winter samples were higher than those in the summer and fall samples. Unfavorable diffusion conditions aggravated the metal pollution in winter, while the low PM_2.5_ mass enhanced the proportion of heavy metals in spring.

### 3.2. Cytotoxicity of PM_2.5_

#### 3.2.1. Cellular ROS

**Figure 5:**
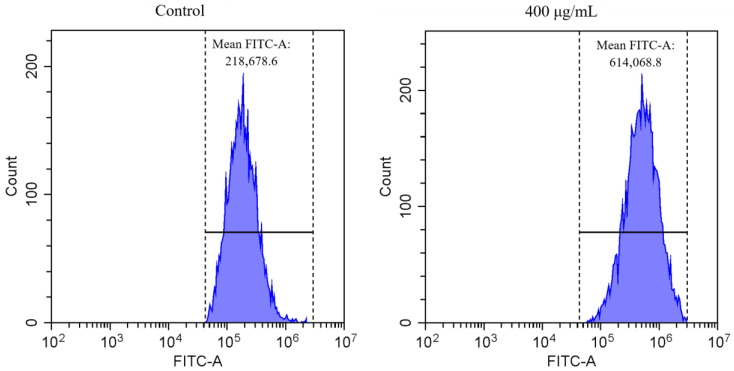
Representative histograms of ROS determination by flow cytometry (the winter sample). Mean FITC-A is the statistical peak area of cell fluorescence stained by the fluorescent probe DCFH-DA.

**Figure 6:**
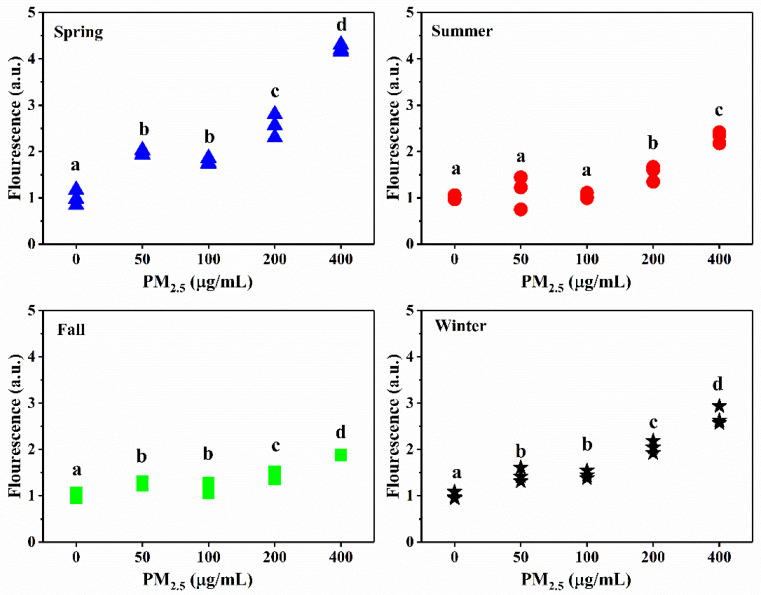
Relative ROS production in the A549 cells exposed to different concentrations of PM_2.5_ Different letters (a, b, c, d) indicate significant differences among the five PM_2.5_ treatments (*p* < 0.05). Each dot is one replicate (*n* = 3).

Oxidative and antioxidant species are in a state of dynamic equilibrium within organisms. When excessive free radicals are produced, such a balance is broken, and oxidative stress and damage may occur. The content of ROS is often used to reflect the degree of oxidative damage caused by PM_2.5_. Previous studies have shown that some water-soluble species (e.g., SO_4_^2^^−^ and NH_4_^+^) in particles had a significant correlation with the level of ROS [49,50]. Some metal elements (e.g., Cr, Mn, Cu, As, Cd, Pb and Zn) have also been demonstrated to induce ROS production, and ultimately cause DNA damage [51,52]. Figure 5 shows the flow cytometry images of the A549 cells with or without PM_2.5_ exposure (from the control treatment and the highest concentration treatment, the winter sample). The statistical results of the ROS levels of the four season samples are shown in Figure 6.

ROS production in all seasons increased with increasing levels of PM_2.5_, suggesting the toxic effects of the particles. Generally, at lower exposure concentrations (50 and 100 μg/mL), there was no significant difference in ROS production for each season (*p >* 0.05). At higher exposure concentrations (200 and 400 μg/mL), a significant increase between the concentration treatments was observed. Moreover, the relative amount of ROS in the spring and winter was higher than those in the summer and fall, indicating stronger oxidative damage in these two seasons. Such variations might be related to the differences in PM_2.5_ composition. Some studies indicated that Zn was the main metal element causing ROS production in A549 cells [49]; other studies also showed that intracellular ROS production was correlated with the metal components (e.g., Fe, Cr, Mn, Ni, Cu, As, Sr, Cd, Cs, Ba, Pb and Zn) in the particles [53]. In this study, the highest Zn content was found in the spring, followed by winter, which might partially explain the observed differences. The seasonal variation of the Pb, Co, Cd, Mn, Cr and Cu contents was basically the same as that of the ROS levels. Additionally, the sum of SO_4_^2−^ and NO_3_^−^ percentages was also higher in the spring and winter, implying their contributions to ROS production [24].

#### 3.2.2. Cell Apoptosis

**Figure 7:**
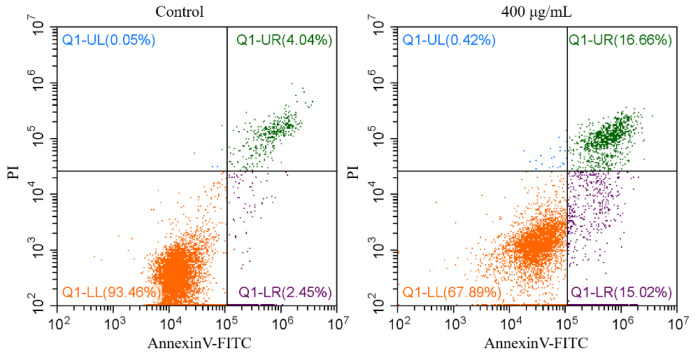
Representative cross quadrant images of Annexin V-FITC/PI double staining determined by flow cytometry (the summer sample).

**Figure 8:**
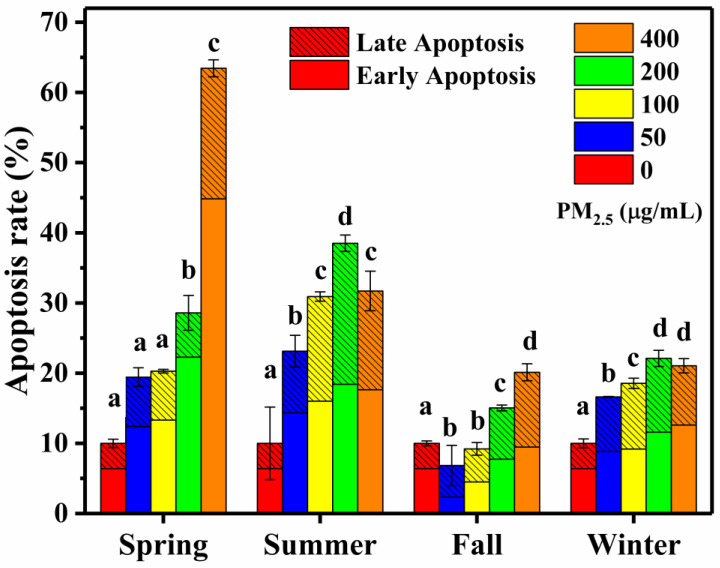
Apoptosis rates in the A549 cells exposed to different concentrations of PM_2.5._ Early apoptosis is the percentage of cells stained by Annexin V, and late apoptosis is the percentage of cells stained by Annexin V and PI. The total apoptosis rate is the percentage of cells in the early and late stages of apoptosis. Different letters (a, b, c, d) indicate significant differences among the five PM_2.5_ treatments in each season (*p* < 0.05). Data are the mean ± SD (*n* = 3).

Apoptosis is a programmed and a non-inflammatory form of cell death within the cells. Many components in PM_2.5_ (e.g., Fe, Cr, Cd and PAHs) have been reported to cause DNA damage and act as inducers of apoptosis in human cells [54]. In addition, the increase in ROS content caused by PM_2.5_ will lead to lipid peroxidation, increase membrane permeability, cause cell dysfunction and eventually apoptosis [19,20]. In this study, Annexin V-FITC/PI double staining reflected early and late apoptosis. Actually, both apoptosis and necrosis can be detected by this staining method. Figure 7 shows the flow cytometry images of the A549 cells with or without PM_2.5_ exposure (from the control treatment and the highest concentration treatment, the summer sample). Early apoptosis is the percentage of cells stained by Annexin V, and late apoptosis is the percentage of cells stained by Annexin V and PI. The total apoptosis rate was then calculated as the percentage of cells in the early and late stages of apoptosis (Figure 8).

The control treatments in the four seasons were maintained at relatively low apoptosis rates (approximately 10%), and there was no significant difference among seasons. At higher levels of PM_2.5_, the apoptosis rates in all seasons generally showed increasing trends. However, decreased apoptosis was found at the highest concentration (400 μg/mL) in the summer and winter samples. During our experiment, the extremely high concentration might have induced cell lysis or suspension, making the cells hard to be collected and labeled, thus leading to an underestimate in apoptosis determination. Although the spring samples had the highest apoptosis rate (63.4 ± 1.4%), at the same dose level, the apoptosis rates of the summer samples were significantly higher than those of the other seasons. Particularly, higher early apoptosis rates in the spring and summer contributed to the higher total apoptosis rates in these seasons. The late apoptosis rates were higher in the summer, followed by winter, spring and fall, which also lead to the higher total apoptosis rates in the summer. Some studies have confirmed the damage effects of organic components on cellular DNA [53]. The specific species were still not clear, but the higher WSOC contents might be related to the higher apoptosis rates in summer.

#### 3.2.3. Correlation between the PM_2.5_ Components and Cytotoxicity

To further quantify the effects of the PM_2.5_ composition on the toxic endpoints, we performed regression analyses between the heavy metals, water-soluble ions, WSOC, intracellular ROS and apoptosis rates. The results are shown in Table 1.

It can be seen that, among all the heavy metals, Zn had the strongest correlation with ROS. This was consistent with our findings that they both had high concentrations in spring and winter. Zn accounts for a large proportion of particulate heavy metals, and it has been demonstrated that Zn in PM_2.5_ could promote the production of ROS [54]. As expected, other metals and the sum of the heavy metals also had strong correlations with ROS. Most of the water-soluble species (e.g., SO_4_^2−^, NO_3_^−^, NH_4_^+^ and WSOC) also showed a strong correlation with ROS, indicating their contribution to ROS production [24]. In addition, Nordin et al. proved that K^+^ was highly related to cellular inflammatory factors, and the inflammatory response would lead to an increase in ROS production [55], which could explain the strong correlation between K^+^ and ROS. As for apoptosis, the correlation between the Zn and apoptosis rate was also the strongest. Excessive Zn^2+^ could indirectly induce the expression of the metallothionein genes (HSP70) and proto-oncogenes (e.g., C-fos, C-jun and C-myc). In addition, high concentration of Zn^2+^ could also decrease the activity of Cu and Zn-superoxide dismutase, increase intracellular free radicals, cause DNA damage and induce apoptosis [20]. Thus, the toxicity of Zn in PM_2.5_ deserves further investigation. 

## 4. Conclusions

PM_2.5_ exposure increased the relative ROS production and apoptosis rates of the A549 cells, and showed obvious seasonal variations, which may be explained by the changes in PM_2.5_ components. Higher ROS production was found in the spring and winter samples, which was consistent with the seasonal changes in some heavy metals and water-soluble species (e.g., NO_3_^−^ and NH_4_^+^). In contrast, Zn and WSOC might play important roles in apoptosis. Of course, the chemical composition of PM_2.5_ is extremely complex, and the relationship between its composition and toxicity is complicated. Therefore, the contributions of different components to PM_2.5_ toxicity and the specific toxic mechanisms of each component deserve further study.

## Figures and Tables

**Table 1:** Correlation coefficients (R) between the ROS/apoptosis and PM_2.5_ composition.

**R**	**As**	**Zn**	**Pb**	**Co**	**Cd**	**Ni**	**Mn**	**Cr**	**Cu**	**Al**
ROS	0.465 *	0.863 *	0.807 *	0.795 *	0.794 *	0.793 *	0.805 *	0.797 *	0.804 *	0.803 *
Apop	0.295	0.675 *	0.624 *	0.612 *	0.612 *	0.618 *	0.612 *	0.611 *	0.611 *	0.596 *
**R**	**Total Metals**	**Cl** **¯**	**SO_4_^2−^**	**NO** **_3_^−^**	**Na** **^+^**	**NH** **_4_^+^**	**K** **^+^**	**Mg** **^2^** **^+^**	**Ca** **^2^** **^+^**	**WSOC**
ROS	0.773 *	0.503 *	0.754 *	0.708 *	0.578 *	0.756 *	0.824 *	0.722 *	0.731 *	0.737 *
Apop	0.586 *	0.345	0.599 *	0.514 *	0.395	0.567 *	0.624 *	0.574 *	0.590 *	0.569 *

ROS: reactive oxygen species; Apop: apoptosis; WSOC: water-soluble organic carbon; * indicates *p <* 0.05.
